# Relationship between low levels of circulating TRAIL and atheromatosis progression in patients with chronic kidney disease

**DOI:** 10.1371/journal.pone.0203716

**Published:** 2018-09-11

**Authors:** Maria Vittoria Arcidiacono, Erika Rimondi, Elisa Maietti, Elisabetta Melloni, Veronica Tisato, Stefania Gallo, Jose Manuel Valdivielso, Elvira Fernández, Àngels Betriu, Rebecca Voltan, Giorgio Zauli, Stefano Volpato, Paola Secchiero

**Affiliations:** 1 Institute for Maternal and Child Health, IRCCS “Burlo Garofolo”, Via dell'Istria, Trieste, Italy; 2 Department of Morphology, Surgery and Experimental Medicine and LTTA Centre, University of Ferrara, Via Fossato di Mortara 70, Ferrara, Italy; 3 Department of Medical Sciences, University of Ferrara, Via Fossato di Mortara 64/b, Ferrara, Italy; 4 Grupo de Investigación Translacional Vascular y Renal, and RedinRen RETIC, ISCIII, Instituto de Investigación Biomédica de Lleida (IRBLleida), Lleida, Spain; International University of Health and Welfare, School of Medicine, JAPAN

## Abstract

**Background:**

Chronic kidney disease (CKD) patients experience a high risk of cardiovascular disease (CV); however, the factors involved in CV-related morbidity and mortality in these patients have not been fully defined. Tumor necrosis factor related apoptosis-inducing ligand (TRAIL) is a cytokine, which exhibits pleiotropic activities on endothelial, vascular smooth muscle and inflammatory cells, with relevant effects on atheromatous plaque formation. On this basis, the present study aims to investigate the role of TRAIL in atheromatosis progression in CKD patients.

**Methods:**

Circulating TRAIL levels were measured in 378 CKD patients belonging to the Spanish National Observatory of Atherosclerosis in Nephrology (NEFRONA) study. All patients were free of previous CV events. Carotid and femoral B-mode ultrasound was performed to detect the presence of plaque at baseline and after 24 months of follow-up.

**Results:**

The lowest levels of TRAIL at baseline were significantly (p<0.05) associated with the appearance, after 24 months of follow-up, of at least two new atheromatous plaques in all territories and of one new plaque in the carotid artery, even after adjusting for CV risk factors. In addition, the patients with low levels of TRAIL at baseline were characterized by the presence of at least one hypoechoic plaque in the carotid artery. This association was significant (p<0.05) even after adjusting for CKD stage.

**Conclusions:**

Overall, the results of our study suggest TRAIL as an assertable independent prognostic biomarker for atheromatosis plaque formation in CKD patients. This observation further supports the potential role of TRAIL for the prevention/treatment of CV disease.

## Introduction

The tumor necrosis factor (TNF)-related apoptosis-inducing ligand (TRAIL) is a member of the TNF superfamily deeply investigated for its ability to induce apoptosis in tumor cells but leaving normal cells virtually unaffected in favour of pro-survival pathways activation [1, 2]. TRAIL mediates several pleiotropic effects by virtue of a complex receptor system including both transmembrane and decoy receptors [3]. Our group has prior investigated the role of TRAIL in pathological conditions such as acute myocardial infarction, diabetes and hypercholesterolemia, and indicated that circulating levels of TRAIL inversely correlate with all-causes and cardiovascular mortality [4–14].

Atheromatosis is an inflammatory disorder of the vessel wall in which endothelial cells (ECs), vascular smooth muscle cells (VSMCs) and inflammatory cells interact with a variety of soluble mediators, including TRAIL. In this setting, TRAIL, which is involved in antagonistic processes, modulating activation, apoptosis and/or necrosis of the vascular and inflammatory cells, exerts either a protective or a pro-atheromatous effect [15]. Indeed, although TRAIL-mediated apoptosis of ECs could promote atheromatosis [16], TRAIL-mediated induction of ECs proliferation and of anti-inflammatory effects, with a reduction of monocyte/leukocyte infiltration into the vessel wall, could exert an overall anti-atherosclerotic effect [17]. A similar controversial activity has been reported on VSMCs where, at early stages, TRAIL-induced apoptosis might favour the atheromatous process [18], while TRAIL-induced proliferation might be beneficial promoting plaque stabilization at late stages [19]. Moreover, in atherosclerotic ApoE-/- mice, TRAIL expression, as well as the expression of its soluble receptor osteoprotegerin (OPG), increased in the aortic valves suffering from an accelerating atheromatous process and vascular calcification, perhaps due to apoptotic bodies derived from VSMCs [20]. On the contrary, in the TRAIL-/-/ApoE-/- mouse model, TRAIL resulted being protective against vascular calcification [21], while in dialysis patients circulating TRAIL levels did not associate with vascular calcification. Moreover, in *in vitro* experiments, recombinant TRAIL was able to induce VSMCs calcification only when cells were treated with phosphate [20].

Accelerated atherosclerosis is a common feature of patients affected by chronic kidney disease (CKD) and these patients are at higher risk of death from cardiovascular disease (CVD) compared with the general population [22]. However, the underlying mechanisms linking CKD to accelerated CVD remain undefined. The predictive value on the progression of plaque formation of either the classical CVD risk factors (e.g. hypertension, diabetes, dyslipidemia, and smoking) or of the disease specific features (e.g. bone mineral dysregulation and inflammation) [23–26] is not completely elucidated and emerging soluble factors seem to contribute to CKD and its complications.

In the light of its controversial implications in the induction/control of the atheromatous process, understanding the role of TRAIL levels in the progression of atheromatosis is of high clinical importance, especially in CKD patients free of cardiovascular events. On these bases, the purpose of the current study was to assess the levels of circulating TRAIL in a population affected by CKD, and to investigate potential association with progression of subclinical atheromatosis in these patients.

## Materials and methods

### Study population

The study population included 378 CKD patients at stages 3 and 4–5 from the multicentre and observational study NEFRONA [27] that consists of a cohort characterized by a high rate of atheromatosis [27–30]. Age ranged between 22 and 76 years. Patients were recruited from October 2009 to June 2011 and followed-up at 24 months. All the included patients had no reported history of CVD (angina pectoris, acute myocardial infarction, ischaemic stroke, haemorrhagic stroke, abdominal aortic aneurysm and atherosclerosis) at baseline. Further exclusion criteria included pregnancy, active infections (HIV and tuberculosis), organ transplantations, previous history of carotid artery disease, hospitalization in the last month, and survival expectation less than 1 year.

The Ethical Committees of all involved Spanish nephrology centres approved this study with the final approval by the Ethics committee board of the Hospital Universitario Arnau de Vilanova (Lleida, Spain). The investigation has been conducted according to the principles expressed in the Declaration of Helsinki, and all the included patients provided written informed consents.

### Clinical and biochemical data

At recruitment, performed three months before the vascular exploration, patients informed about their medical history including diabetes, hypertension, dyslipidemia, and about their lifestyle habits including smoking and drinking. At the same time, blood was drawn at fasting. Serum samples were then stored at the centralized biobank of the Spanish Network for Nephrology Research (REDinREN). Biochemical analysis were performed as described in Betriu et al. [27], while morphometric parameters were measured as in Arcidiacono et al. [31]. Serum TRAIL concentration was measured with the commercially available Quantikine^®^ ELISA kit (# DTRL00, R&D Systems) according to the manufacturer’s instructions as previously described [32, 33].

### Carotid and femoral ultrasound

Assessment of the presence and composition of atheromatous plaques was carried out by carotid and femoral B-mode ultrasound analyses performed at baseline and at 24 months from the inclusion in the study. A Vivid BT09 Apparatus (GE Healthcare, Waukesha, WI) equipped with a 6-13MHz broadband linear array probe was used for this assay. Three segments (common, bulb and internal) of both carotid arteries were explored with the patients in a supine position and the head 45° angled contralateral to the side of the probe. Both common and superficial femoral arteries were also explored by ultrasound.

In a blinded fashion, a unique reader defined the presence of atheromatous plaques, which were defined as intima media thickness > 1.5 mm [34, 35], and classified as predominantly lipidic (type 1 and 2), fibrotic (type 3 and 4) or calcified (type 5) in accordance with their echogenicity. The same itinerant team performed the ultrasound exploration, and only one reader evaluated the ultrasound images.

## Statistical analysis

Results were reported as mean±SD for continuous variables with approximate normal distribution, while median and inter-quartile range ([IQR]) were used to describe the other numerical variables, instead counts and percentages were used for categorical characters. For analytical purpose, TRAIL was stratified by tertiles. Comparisons between groups were done using chi-squared test for categorical variables, while for continuous ones, the ANOVA, the t-test on means, or the non-parametric Kruskal Wallis and the Wilcoxon-Mann-Whitney tests were used as appropriate. A first analysis evaluated all the factors that were related to TRAIL and the progression of the atheromatosis process. Consequently, a regression analysis was performed including all the possible confounding factors that resulted associated to both TRAIL and outcomes (increasing of plaque number in the carotid and femoral arteries). Poisson regression was estimated for the outcome “number of new plaques” while logistic regression model was used for the other dichotomous outcomes. Five nested models were built: simple, stage adjusted, adjusted for stage and demographics variables, additional adjustment for diabetes and statins treatment. Statistical analysis was done using Stata 13.0 (Stata Corp, College Station, TX) statistical program, with a significance level of 0.05.

## Results

### Association of TRAIL levels with baseline characteristics of the study population

**Fig 1:**
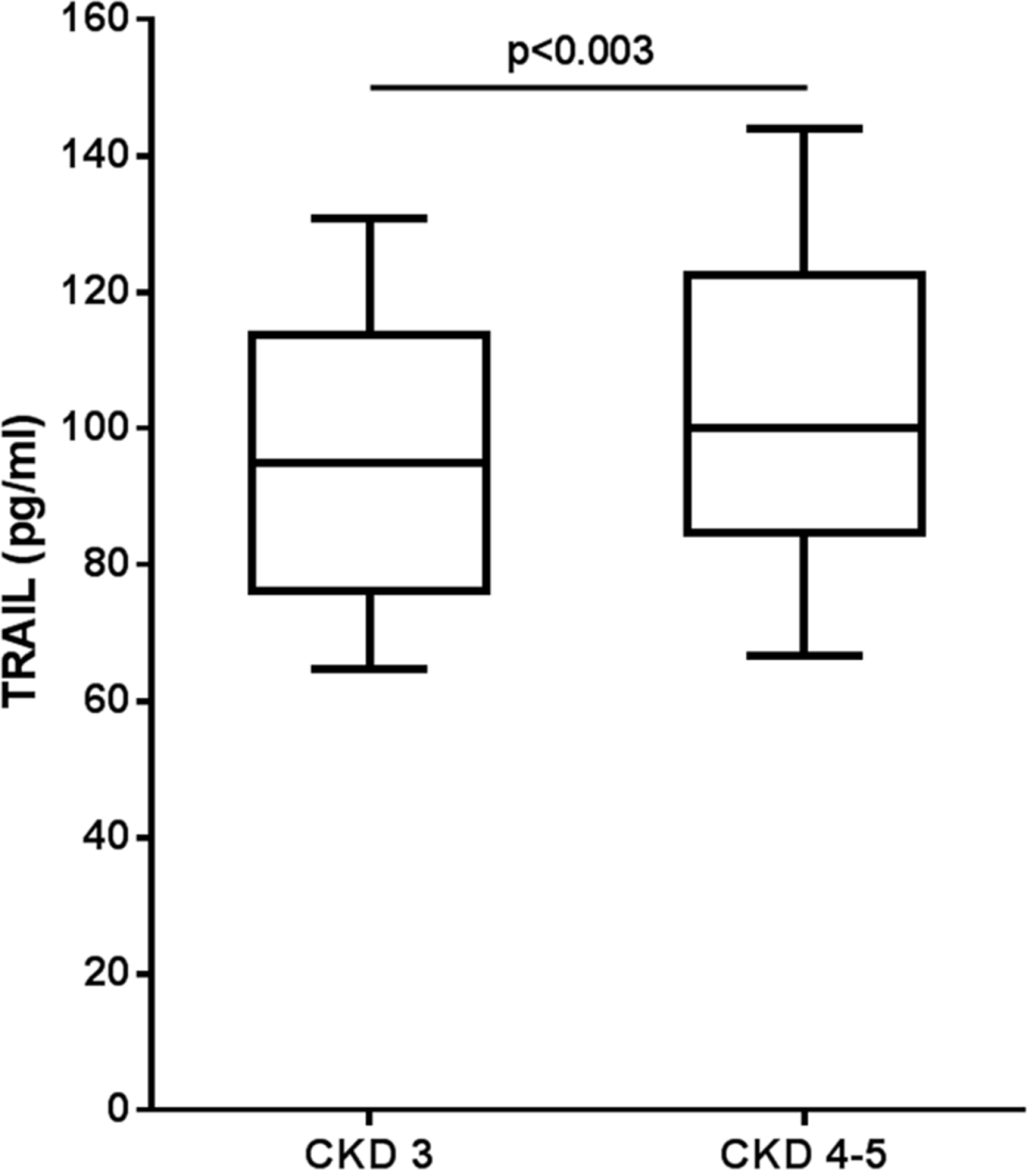
TRAIL levels stratified by CKD stages at baseline. Box plot graphs showing the levels of TRAIL in patients at CKD 3 or CKD 4–5 stages. Levels of TRAIL are expressed as median [IQR], minimum and maximum.

For the purpose of our study, we have assessed 378 CKD patients, at stages 3 and 4–5, from the multicentre and observational study NEFRONA. This cohort was chosen because all the included patients had no reported history of CVD at baseline and therefore represents the best condition for the identification of predictive markers of CV events. As shown in Fig 1, at baseline, the circulating levels of TRAIL were significantly (*p* = 0.003) higher in patients at CKD stage 4–5 compared to those in patients at CKD stage 3. Based on this difference in TRAIL levels, we further investigated the association of TRAIL with all the demographic, clinical, morphometric and biochemical variables (as reported in S1 Table). As expected [32], diabetes prevalence was significantly (*p* = 0.023) higher in patients with the lowest levels of TRAIL. In addition, the percentage of patients under statins treatment and the levels of uric acid were statistically higher in the highest tertile of TRAIL (*p* = 0.021 and *p* = 0.001, respectively). According to the lifestyle habit, smokers were characterized by the lowest levels of TRAIL.

### Low TRAIL levels at baseline were associated with higher atherosclerosis progression

**Fig 2:**
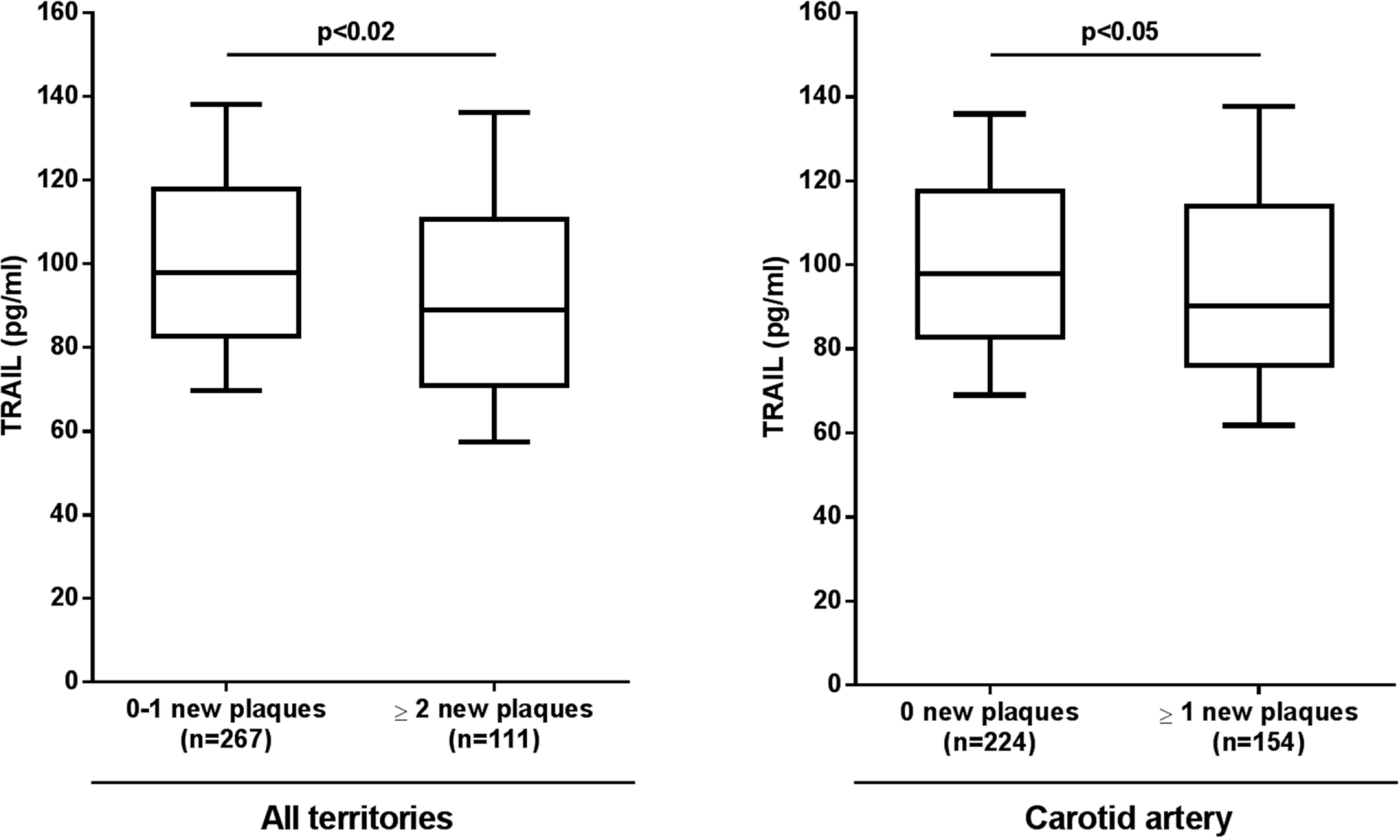
Association between circulating TRAIL levels and the presence of new atheromatous plaques in all territories and in the carotid artery only. Box plot graphs showing the levels of TRAIL in patients with or without two new plaques in all territories (carotid and femoral arteries, left panel), or in patients with or without one new plaque in the carotid artery (right panel) after 24-months follow-up. Levels of TRAIL are expressed as median [IQR], minimum and maximum.

In the next set of analyses, we investigated the association of TRAIL levels at baseline with atheromatosis progression, assessed in all patients at 24 months follow-up. At baseline, no differences in the number of plaques were observed according to the levels of TRAIL. On the contrary, after 24 months-follow up the patients with at least two new plaques in all territories (*p*<0.02) and one new plaque in the carotid artery (*p*<0.05) showed the lowest levels of TRAIL (Fig 2).

Moreover, patients with at least four or more plaques had even lower levels of TRAIL at baseline (*p* = 0.033). As expected, additional factors were significantly (*p*<0.05) associated with the onset of two or more new plaques at 24 months-follow up (Table 1), such as being older and male, suffering from diabetes, having high systolic blood pressure (SBP), triglycerides (TG), C-reactive protein (CRP), and high cigarettes and alcohol consumption. In addition, the lack of statin treatment resulted in two or more new plaques (*p*<0.05).

**Table 1:** Clinical, biochemical and morphometric variables associated with the development of at least two new atheromatous plaques.

	0–1 new plaque (n = 267)	≥2 new plaques (n = 111)	*p*-value
**Demographic variables**			
Age (years)	57.3 ± 12.6	61.8 ± 9.8	**<0.001**
Male (%)	152 (56.9)	76 (68.5)	**0.037**
**Clinical variables**			
Diabetes (%)	64 (20.0)	43 (38.7)	**0.004**
CKD stage 4–5 (%)	113 (42.3)	55 (49.6)	0.198
Hypertension (%)	245 (91.8)	102 (91.9)	0.966
Dyslipidemia (%)	175 (65.5)	76 (68.5)	0.583
**Morphometric and biochemical variables**			
BMI (Kg/m^2^)	28.9 ± 4.9	29.8 ± 5.4	0.106
SBP (mmHg)	138 ± 18	146 ± 20	**<0.001**
HDL cholesterol (mg/dL)	50.7 ± 14.1	48.2 ± 15.1	0.148
LDL cholesterol (mg/dL)	105.9 ± 32.0	110.7 ± 32.8	0.215
TG (mg/dL)^a^	119 [88–169]	142 [107–170]	**0.023**
Creatinine (mg/dL)	2.25 ± 1.09	2.39 ± 1.02	0.267
Glomerular filtration rate (mL/min)	34.4 ± 13.5	32.3 ± 13.1	0.167
Uric acid (mg/dL)	7.1 ± 1.6	7.0 ± 1.7	0.588
Phosphorus (mg/dL)^a^	3.7 [3.2–4.1]	3.6 [3.2–4.1]	0.933
Ferritin (mg/dL)^a^	123 [62–216]	114 [58–260]	0.798
CRP (mg/L)^a^	1.9 [1.0–3.8]	2.9 [1.3–6.4]	**0.005**
25(OH)-vitamin D3 (ng/L)	18.1 ± 7.9	16.5 ± 6.6	0.056
Hemoglobin (g/dL)	13.2 ± 1.6	13.2 ± 1.9	0.792
**Treatments**			
Statins treatment (%)	159 (59.6)	53 (47.8)	**0.035**
Antipertensive (%)	249 (93.3)	108 (97.3)	0.118
**Lifestyle habits**			
Smoking status (Current or Former) (%)	140 (52.4)	78 (70.3)	**0.001**
Alcohol			**0.004**
>0 <10g	76 (28.5)	23 (20.7)	
10<20g	19 (7.1)	20 (18.0)	
> = 20g	22 (8.2)	14 (12.6)	

^a^Identified variables with significant deviations from normal distribution. Values of these variables are provided as median [IQR]. For variables with normal distribution, values are expressed with mean±SD. BMI: body mass index; SBP: systolic blood pressure; TG: triglycerides; CKD: Chronic Kidney Disease; CRP: C-reactive protein.

Therefore, in order to establish if the level of TRAIL at baseline could be an independent biomarker of atheromatosis progression, five regression models, including each different set of confounding factors associated to both TRAIL and atheromatosis progression were performed as described in Table 2. Importantly, in comparison with the simple model (OR = 0.72 (0.57–0.92)), even after adjusting for CKD stage and demographic variables (age, sex and smoking status), low levels of TRAIL resulted independently associated with the formation of two or more new plaques (OR = 0.75 (0.59–0.96), *p* = 0.025). Similar results were observed even when adjusting for statin treatment only, or adjusting for statin treatment and diabetes further supporting the association between circulating TRAIL levels and the formation of at least two new plaques in all territories (OR = 0.77 (0.60–0.99), p = 0.046 and OR of 0.78 (0.61–1.00), *p* = 0.054, respectively).

**Table 2:** Soluble TRAIL effects on atheromatous progression.

		Simple model	Stage adjusted model	Stage and demographic^a^ adjusted model	Full adjusted model^b^	Full adjusted model^c^
**Number of new plaques in all territories**^c^	IRR (95% CI)	0.86 (0.78–0.95)	0.85 (0.77–0.94)	0.80 (0.81–0.99)	0.91 (0.82–1.01)	0.91 (0.82–1.01)
	*p*-value	**0.003**	**0.001**	**0.036**	0.072	0.084
**Presence of at least 2 new plaques in all territories**^d^	OR (95% CI)	0.72 (0.57–0.92)	0.70 (0.55–0.89)	0.75 (0.59–0.96)	0.77 (0.60–0.99)	0.78 (0.61–1.00)
	*p*-value	**0.008**	**0.004**	**0.025**	**0.046**	**0.054**
**Presence of at least 1 new plaque in the carotid artery**	OR (95% CI)	0.80 (0.65–0.99)	0.78 (0.63–0.97)	0.83 (0.66–1.04)	0.82 (0.66–1.03)	0.83 (0.66–1.04)
	*p*-value	**0.041**	**0.025**	0.099	0.094	0.106

^a^age, sex and smoking status.

^b^stage and demographic variables plus statins treatment.

^c^stage and demographic variables plus diabetes and statins treatment.

^d^All territories means carotid and femoral arteries.

Lastly, considering the instability and the role of hypoechoic plaques in contributing to a higher incidence of CV events, a further analysis explored TRAIL levels in correlation with plaque composition. Specifically, the lowest levels of TRAIL were observed in patients with at least one new hypoechoic plaque after 24 months-follow up in the carotid artery (*p* = 0.013), even after adjusting for CKD stage (*p* = 0.015), a well-recognized factor for plaque composition.

## Discussion

In the present study, conducted on patients affected by CKD at stages 3 to 5 without previous cardiovascular events, we demonstrated an independent inversely association between the levels of TRAIL and atheromatosis progression. Indeed, low levels of TRAIL statistically significant associated with atheromatosis progression even after adjusting for the well-recognized risk factors for atheromatosis, such as CKD stage, age, sex, and smoking status [27]. Moreover, the adjustment for diabetes and statin treatments, two established modulators of atheromatosis progression [30], confirmed that low levels of circulating TRAIL associated to the appearance of at least two new atheromatous plaques after 24-months of follow-up.

These results further support the involvement of TRAIL on cardiovascular disease since they are in agreement with previous studies demonstrating that the levels of TRAIL are reduced in patients with acute coronary syndromes [6, 36], congestive heart failure [37] and in a CKD population with an elevated risk of mortality [7]. Moreover, our observations indicate a role of TRAIL as a prognostic factor in CKD patients without previous cardiovascular events [4].

Although the precise mechanism by which low levels of TRAIL affect the atheromatosis progression is still unknown, we postulate some potential mechanisms. A first mechanism could involve VSMCs apoptosis or senescence that has been recently demonstrated promoting both plaque formation and instability [38]. In fact, previous *in vitro* experiments demonstrated that treatment with recombinant TRAIL protected VSMCs from apoptosis [39]. Moreover, in the ApoE-/- mouse model the administration of recombinant TRAIL led to VSMCs proliferation reducing atheromatosis progression [19]. A further conceivable explanation that cannot be ruled out is the role of TRAIL in counteracting the proadhesive activity of TNF-α [40]. In this respect, circulating TRAIL reduction could be the cause of a higher leucocyte adhesion and infiltration through the endothelial layer leading to atheromatous progression. Importantly, these data are in part supported by the inverse correlation of the levels of TRAIL and the presence of at least one unstable hypoechoic plaque in the carotid artery. Indeed, hypoechoic plaques are characterized by high prevalence of leucocytes and foam cells and low prevalence of VSMCs.

Intriguing is the correlation of incidence of diabetes, smoking, statin treatment and CKD stage with TRAIL levels. Indeed our results are in accordance with the data of Di Bartolo et al. [41], in which the incidence of diabetes inversely correlated with TRAIL levels. Moreover, the concurrence of a low incidence of statin treatment with low levels of TRAIL, and their role on the development of new atheromatous plaques, indicate either that there is a synergic effect on plaque formation [42], or that low levels of TRAIL at baseline are a marker of inadequate treatment of dyslipidemia. In fact, *in vitro* and *in vivo* studies demonstrated that the lack of TRAIL increases the levels of cholesterol [41]. The latter hypothesis could improve the managing of dyslipidemia in CKD at stages 3 to 5 [43]. In addition, although previous mechanistic demonstrations are unavailable, the inverse association between smoking status and TRAIL leads to suppose that smoking habit, which is a well-known CV risk factor, could be involved in the major stages of atherosclerosis through TRAIL modulation. Moreover, higher levels of TRAIL at baseline in the CKD4-5 population with respect to the CKD3 population could indicate the worst renal condition of these patients at recruitment, as postulated by Cartland et al. [44]. Nevertheless, in the prospective analysis at 24-months follow up, low levels of TRAIL at baseline resulted an independent factor for atheromatosis progression. Similar results were observed in a population with acute myocardial infarction in which only the low levels of TRAIL at hospitalization, rather than the normal levels after 12-month of follow up, correlated with a higher incidence of cardiovascular events [6].

Our findings indicate TRAIL as a prognostic factor that deserves further investigation in larger cohorts of CKD patients at different stages and with a longer follow-up. Certainly, the strengths of the present study comprise: i) the inclusion of only CKD patients at stages 3 and 4–5 from daily clinical care, resulting in a representative population with the highest atheromatous mortality risk [27]; ii) the ultrasound exploration done by the same team and evaluated by the same reader; iii) precise and unique information on plaque morphology.

## Conclusions

This work provides the evidence that the level of TRAIL at baseline is an independent prognostic factor for novel atheromatous plaque formation in CKD patients at stages 3 to 5. From a therapeutic point of view, these results establish the basis for the evaluation of the role of soluble recombinant TRAIL in preventing/attenuating atheromatous progression in CKD patients not undergoing dialysis.

## Supporting information

S1 Table Baseline characteristics of the study population according to TRAIL tertile distribution. (PDF)

S1 Appendix List of the NEFRONA investigators. (PDF)
