# Supplementary material for: Relationship between low levels of circulating TRAIL and atheromatosis progression in patients with chronic kidney disease
Source: PLoS One. 2018 Sep 11;13(9):e0203716. doi: 10.1371/journal.pone.0203716 (PMC6133360; doi:10.1371/journal.pone.0203716)
Supplement: S1 Table — (PDF) [file pone.0203716.s001.pdf]

S1 Table. Baseline characteristics of the study population according to TRAIL tertile distribution.

	TRAIL tertiles (pg/ml)			
	[15.5 – 85.6)<br>(n= 126)	[85.6 – 108.0)<br>(n= 126)	[108.0 -200.8]<br>(n= 126)	*p*-value
**Demographic variables**				
Age (years)	60.5 ± 12.1	58.0 ± 11.7	57.4 ± 12.3	0.261
Male (%)	79 (62.7)	79 (61.9)	71 (56.3)	0.533
**Clinical variables**				
Diabetes (%)	44 (34.9)	25 (19.8)	38 (30.2)	**0.023**
Hypertension (%)	116 (92.1)	112 (88.9)	119 (94.4)	0.272
Dyslipidemia (%)	80 (63.5)	83 (65.9)	88 (69.8)	0.559
**Morphometric and biochemical variables**				
BMI (Kg/m^2)	29.1 ± 5.5	29.0 ± 4.9	29.5 ± 4.8	0.761
SBP (mmHg)	141 ± 20	140 ± 19	141 ± 18	0.753
HDL cholesterol (mg/dL)	49.0 ± 15.4	50.2 ± 13.9	50.6 ± 14.1	0.712
LDL cholesterol (mg/dL)	104.5 ± 31.3	111.8 ± 34.5	105.4 ± 30.6	0.175
TG (mg/dL)[a]	129 [95 – 171]	125 [87 – 165]	130 [92 – 166]	0.664
Creatinine (mg/dL)	2.16 ± 0.94	2.37 ± 1.10	2.34 ± 1.14	0.243
Glomerular filtration rate[a] (mL/min)	35.6 ± 13.2	33.0 ± 13.6	32.7 ± 13.3	0.179
Uric acid (mg/dL)	6.6 ± 1.6	7.2 ± 1.7	7.3 ± 1.5	**0.001**
Phosphorus (mg/dL)[a]	3.7 [3.3 – 4.1]	3.5 [3.2 – 4.1]	3.7 [3.3 – 4.2]	0.307
Ferritin (mg/dL)[a]	104 [70 – 223]	137 [52 – 229]	132 [67 – 239]	0.814
CRP (mg/L)[a]	2.3 [1.0 – 5.4]	1.8 [1.1 – 3.9]	2.1 [1.2 – 4.1]	0.404
25(OH)-vitamin D3 (ng/L)	16.7 ± 7.6	18.5 ± 7.2	17.6 ± 8.0	0.176
Hemoglobin (g/dL)	13.2 ± 1.6	13.3 ± 1.8	13.2 ± 1.6	0.827
**Treatments**				
Statins treatment (%)	62 (49.2)	67 (53.2)	83 (65.9)	**0.021**
Antipertensive (%)	118 (93.7)	117 (92.9)	122 (96.8)	0.347
**Lifestyle habits**				
Smoking status (Current or Former) (%)	83 (65.9)	70 (55.6)	65 (51.6)	0.062
Alcohol<br>>0 <10g<br>10<20g<br>>=20g	 28 (22.2)<br>14 (11.1)<br>13 (11.3)	 37 (29.4)<br>14 (11.1)<br>19 (13.9)	 34 (27.0)<br>11 (8.7)<br>8 (5.8)	0.313

[a]Identified variables with significant deviations from normal distribution. Values of these variables are provided as median [IQR]. For variables with normal distribution values are expressed with mean±SD. BMI: body mass index; SBP: systolic blood pressure; TG: triglycerides; CKD: Chronic Kidney Disease; CRP: C-reactive protein.
