# Supplementary material for: Relationship between low levels of circulating TRAIL and atheromatosis progression in patients with chronic kidney disease
Source: PLoS One. 2018 Sep 11;13(9):e0203716. doi: 10.1371/journal.pone.0203716 (PMC6133360; doi:10.1371/journal.pone.0203716)
Supplement: S1 Appendix — (PDF) [file pone.0203716.s002.pdf]

NEFRONA investigators: Aladrén Regidor, Mª José. Hospital Comarcal Ernest Lluch (Calatayud); Almirall, Jaume; Ponz, Esther. Corporació Parc Taulí (Barcelona); Arteaga Coloma, Jesús. Hospital de Navarra (Pamplona); Bajo Rubio, Mª Auxiliadora; Díaz, Raquel Raquel Hospital La Paz (Madrid); Belart Rodríguez, Montserrat. Sistemes Renals (Lleida); Gascón, Antonio, Hospital Obispo Polanco (Teruel); Bover Sanjuan, Jordi. Fundació Puigvert. IIB Sant Pau (Barcelona); Bronsoms Artero, Josep. Clínica Girona (Girona); Cabezuelo Romero, Juan B; Muray Cases, Salomé. Hospital Reina Sofía (Murcia); Calviño Varela, Jesús. Hospital Universitario Lugus Augusti (Lugo); Caro Acevedo, Pilar. Clínica Ruber (Madrid); Carreras Bassa, Jordi. Diaverum Baix Llobregat (Barcelona); Cases Amenós, Aleix; Massó Jiménez, Elisabet. Hospital Clínic (Barcelona); Moreno López, Rosario. Hospital de la Defensa (Zaragoza); Cigarrán Guldris, Secundino; López Prieto, Saray. Hospital Da Costa (Lugo); Comas Mongay, Lourdes. Hospital General de Vic (Barcelona); Comerma, Isabel. Hospital General de Manresa (Barcelona); Compte Jové, Mª Teresa, Hospital Santa Creu Jesús (Tarragona); Cuberes Izquierdo, Marta. Hospital Reina Sofía (Navarra); de Álvaro, Fernando; Hevia Ojanguren, Covadonga. Hospital Infanta Sofía (Madrid); de Arriba de la Fuente, Gabriel. Hospital Universitario Guadalajara (Guadalajara); del Pino y Pino, Mª Dolores. Complejo Hospitalario Universitario Torrecardenas (Almería); Diaz-Tejeiro Izquierdo, Rafael; Ahijado Hormigos, Francisco Hospital Virgen de la Salud (Toledo); Dotori, Marta. USP Marbella (Málaga); Duarte, Verónica. Hospital de Terrassa (Barcelona); Estupiñan Torres, Sara. Hospital Universitario Canarias (Santa Cruz de Tenerife); Fernández Reyes, Mª José. Hospital de Segovia (Segovia); Fernández Rodríguez, Mª Loreto. Hospital Príncipe de Asturias (Madrid); Fernández, Guillermina. Clínica Santa Isabel (Sevilla); Galán Serrano, Antonio. Hospital General Universitario de Valencia (Valencia); García Cantón, Cesar. Hospital Universitario Insular de Gran Canaria (Las Palmas); García Herrera, Antonio L. Hospital Universitario Puerto Real (Cádiz); García Mena, Mercedes. Hospital San Juan de Dios (Zaragoza); Gil Sacaluga, Luis; Aguilar, Maria. Hospital Virgen del Rocío (Sevilla); Górriz, José Luis. Hospital Universitario Doctor Peset (Valencia); Huarte Loza, Emma. Hospital San Pedro (Logroño); Lerma, José Luis. Hospital Universitario Salamanca (Salamanca); Liebana Cañada, Antonio. Hospital de Jaén (Jaén); Marín Álvarez, Jesús Pedro. Hospital San Pedro de Alcántara (Cáceres); Martín

Alemany, Nàdia. Hospital Jose p Trueta (Girona); Martín García, Jesús. Hospital Nuestra Señora de Sonsoles (Ávila); Martínez Castelao, Alberto. Hospital Universitari de Bellvitge (Barcelona); Martínez Villaescusa, María. Complejo Hospitalario Universitario de Albacete (Albacete); Martínez, Isabel. Hospital Galdakao (Bilbao); Moina Eguren, Iñigo. Hospital Basurto (Bilbao); Moreno Los Huertos, Silvia. Hospital Santa Bárbara (Soria); Mouzo Mirco, Ricardo. Hospital El Bierzo, Ponferrada (León); Munar Vila, Antonia. Hospital Universitari Son Espases (Palma de Mallorca); Muñoz Díaz, Ana Beatriz. Hospital Virgen del Consuelo (Valencia); Navarro González, Juan F. Hospital Universitario Nuestra Señora de Candelaria (Santa Cruz de Tenerife); Nieto, Javier; Carreño, Agustín. Hospital General Universitario de Ciudad Real (Ciudad Real); Novoa Fernández, Enrique. Complexo Hospitalario de Ourense (Ourense); Ortiz, Alberto; Fernandez, Beatriz. IIS-Fundación Jiménez Díaz (Madrid); Paraíso, Vicente. Hospital Universitario del Henares (Madrid); Pérez Fontán, Miguel. Complejo Hospitalario Universitario A Coruña (A Coruña); Peris Domingo, Ana. Hospital Francesc de Borja (Valencia); Piñera Haces, Celestino. Hospital Universitario Marqués de Valdecilla (Santander); Prados Garrido, Mª Dolores. Hospital Universitario San Cecilio (Granada); Prieto Velasco, Mario. Hospital de León (León); Puig Marí, Carmina. Hospital d'Igualada (Barcelona); Rivera Gorrín, Maite. Hospital Universitario Ramón y Cajal (Madrid); Rubio, Esther. Hospital Puerta del Hierro (Madrid); Ruiz, Pilar. Hospital Sant Joan Despí Moisès Broggi (Barcelona); Salgueira Lazo, Mercedes; Martínez Puerto, Ana Isabel. Hospital Virgen Macarena (Sevilla); Sánchez Tomero, José Antonio. Hospital Universitario de la Princesa (Madrid); Sánchez, José Emilio. Hospital Universitario Central de Asturias (Oviedo); Sans Lorman, Ramon. Hospital de Figueres (Girona); Saracho, Ramon. Hospital de Santiago (Vitoria); Sarrias, Maria; Serón, Daniel. Hospital Universitari Vall d'Hebron (Barcelona); Soler, María José; Barrios, Clara. Hospital del Mar (Barcelona); Sousa, Fernando. Hospital Rio Carrión (Palencia); Toran, Daniel. Hospital General de Jerez (Cadiz); Tornero Molina, Fernando. Hospital de Sureste (Arganda del Rey); Usón Carrasco, José Javier. Hospital Virgen de la Luz (Cuenca); Valera Cortes, Ildefonso. Hospital Virgen de la Victoria (Málaga); Vilaprinyo del Perugia, Mª Merce. Institut Catala d'Urologia i Nefrologia (Barcelona); Virto Ruiz, Rafael C. Hospital San Jorge (Huesca); Vicente Pallarés Carratalá Clinica MEDEFIS (Vila-real. Castellón), Carlos Santos Altozano CS Azuqueca de

Henares (Guadalajara); Miguel Artigao Ródenas CS Zona III (Albacete); Inés Gil Gil Área Básica Sanitaria de Arán. CAP Viella (Lleida); Francisco Adan Gil CS Alfaro (La Rioja); Emilio García Criado Centro de Salud del Carpio (Córdoba.); Rafael Durá Belinchón CS Godella (Valencia); Jose Mª Fernández Toro CS Zona Centro (Cáceres); Juan Antonio Divisón Garrote Centro de Salud de Casas Ibáñez. Consultorio de Fuentealbilla (Albacete).
